# Item usage in a multidimensional computerized adaptive test (MCAT) measuring health-related quality of life

**DOI:** 10.1007/s11136-017-1624-3

**Published:** 2017-06-23

**Authors:** Muirne C. S. Paap, Karel A. Kroeze, Caroline B. Terwee, Job van der Palen, Bernard P. Veldkamp

**Affiliations:** 1 Department of Special Needs, Education, and Youth Care, Faculty of Behavioural and Social Sciences, University of Groningen, Grote Rozenstraat 38, 9712 TJ Groningen, The Netherlands; 2 Centre for Educational Measurement at the University of Oslo (CEMO), Faculty of Educational Sciences, University of Oslo, Oslo, Norway; 3 Department of Research Methodology, Measurement, and Data-Analysis, Faculty of Behavioural, Management and Social Sciences, University of Twente, Enschede, The Netherlands; 4 Department of Epidemiology and Biostatistics and the EMGO Institute for Health and Care Research, VU University Medical Center, Amsterdam, The Netherlands; 5 Medical School Twente, Medisch Spectrum Twente, Enschede, The Netherlands

**Keywords:** Item exposure, HRQL, IRT, Item response theory, MCAT, CAT, MAT, Computerized adaptive test

## Abstract

**Purpose:**

Examining item usage is an important step in evaluating the performance of a computerized adaptive test (CAT). We study item usage for a newly developed multidimensional CAT which draws items from three PROMIS domains, as well as a disease-specific one.

**Methods:**

The multidimensional item bank used in the current study contained 194 items from four domains: the PROMIS domains fatigue, physical function, and ability to participate in social roles and activities, and a disease-specific domain (the COPD-SIB). The item bank was calibrated using the multidimensional graded response model and data of 795 patients with chronic obstructive pulmonary disease. To evaluate the item usage rates of all individual items in our item bank, CAT simulations were performed on responses generated based on a multivariate uniform distribution. The outcome variables included active bank size and item overuse (usage rate larger than the expected item usage rate).

**Results:**

For average *θ*-values, the overall active bank size was 9–10%; this number quickly increased as *θ*-values became more extreme. For values of −2 and +2, the overall active bank size equaled 39–40%. There was 78% overlap between overused items and active bank size for average *θ*-values. For more extreme *θ*-values, the overused items made up a much smaller part of the active bank size: here the overlap was only 35%.

**Conclusions:**

Our results strengthen the claim that relatively short item banks may suffice when using polytomous items (and no content constraints/exposure control mechanisms), especially when using MCAT.

**Electronic supplementary material:**

The online version of this article (doi:10.1007/s11136-017-1624-3) contains supplementary material, which is available to authorized users.

## Introduction

In the last decade, computerized adaptive tests (CATs) [1] based on item response theory (IRT) [2] have become increasingly popular in health measurement. A CAT can be seen as a questionnaire that is tailored to the test-taker on the fly: it continuously updates the estimate(s) of the position on the construct of interest (*latent trait*) based on answers given by the test-taker to the questions (items) posed. The underlying algorithm then selects the item that is most informative at that particular moment, given the current estimate of the latent trait value. It is clear why CATs appeal to healthcare professionals (HCPs): by selecting only those items that contribute most to the reliable measurement of a patient’s latent trait value, measurement efficiency is increased, which results in a substantial decrease in response burden [3]. Furthermore, CAT estimates can be used to generate automatic reports instantly, providing the HCP with all necessary information (latent trait estimate, standard error, norms, and graphic display) to facilitate communication with the patient. These properties make CATs excellent candidates for monitoring patients’ physical and mental health routinely, be it on a monthly or daily basis.

CATs draw their items from item banks: large collections of items that have been calibrated with an IRT model using a large sample representative of the target population. The quality of the CAT and the latent trait estimate it generates depend to a large degree on the quality of the item bank. A psychometrically sound item bank contains items with location parameters that cover the whole range of relevant latent trait values, while having adequate to high discrimination parameters. A CAT drawing items from such an item bank will result in efficient measurement for all patients (irrespective of their latent trait score). Most CATs currently used for health measurement are based on item banks that were calibrated using unidimensional IRT models (e.g., [4–7]). Although less frequently used, multidimensional IRT models are available as well, and can be used to support multidimensional CAT (MCAT) (e.g., [8–10]). It has been shown that test length can be further reduced by taking the correlation among constructs into account during item selection and latent trait estimation, while maintaining adequate levels of measurement precision [11, 12]. Perhaps equally important, patients often experience quality-of-life (QoL) domains as interdependent; taking this into account allows a closer alignment between psychometric modeling and patient perspective.

Since health-related quality of life (HRQL) has taken a central role in the evaluation of treatment interventions in patients with chronic obstructive pulmonary disease (COPD), we recently developed a multidimensional CAT (MCAT) to measure HRQL in patients with chronic obstructive pulmonary disease (COPD) [13]. Following the steps outlined by Paap et al. [14], we first established which domains of HRQL are most important to patients with COPD, using relevant literature (articles and existing questionnaires), as well as interviews with patients and HCPs [14, 15]. Based on these findings, three generic domains/item banks from the PROMIS (Patient-Reported Outcomes Measurement Information System) framework were selected (fatigue, physical functioning, and ability to participate in social roles and activities) and a new COPD-specific domain/item bank (COPD-SIB) was developed [16]. This approach ensures comparability with other patient groups (generic domains), while providing additional sensitivity for measuring change within the specific patient group (disease-specific domain). In this paper, we aim to evaluate an important performance measure for our CAT: *item usage*.

Due to the adaptive nature of a CAT, it can be expected that certain items are used more frequently than others. Successive items are typically chosen to optimize an objective function [17], such as the Fisher information function.^1^ Highly discriminating items, polytomous items covering a wide range of the latent trait (denoted *θ*), and items targeting average *θ*-values have a higher chance of being selected, all else being equal. If items are selected more frequently than could be expected based on chance or a predefined threshold, these items are typically referred to as being overexposed. Conversely, items selected less frequently than could be expected are referred to as underexposed. The terms *item exposure* and *item usage* seem to be used interchangeably in the literature. In the context of educational testing, item overexposure is seen as a threat to test security (examinees may be able to remember and share items with others) and receives a lot of attention in the literature (see, e.g., [18, 20, 21]); in health measurement, items do not need to be kept secret and therefore item exposure has received less attention [22]. However, item usage is an important outcome measure in evaluating CAT and item bank performance. Variability in item usage rates indicates that the CAT is working as intended (if the items were selected at random, the item usage rate would be expected to be equal for all items). However, if a number of items are not used at all, or very rarely, the “real” (active) size of the item bank is smaller than it was designed to be. The main aim of the current study is to evaluate item usage for a newly developed MCAT which draws items from the PROMIS domains fatigue, physical function, and ability to participate in social roles and activities, as well as the COPD-SIB. We will report on both active bank size and item overuse/overexposure.

## Methods

### Multidimensional item bank

Adams et al. [23] divide multidimensional IRT models into two subclasses: *within-item* and *between-item* multidimensional models. Within-item multidimensional models allow items to relate to more than one latent dimension. When between-item multidimensional models are used, the restriction is imposed that the items relate to one dimension only; multidimensionality is expressed through the correlations among the latent dimensions (these are estimated jointly with the item parameters and latent trait values). In this study, we chose to use a between-item multidimensional model, since such models are useful when multiple distinct latent dimensions are measured^2^ and relatively high correlations are expected. The multidimensional item bank used in the current study contained 194 items from four domains: the PROMIS domains fatigue (example item: “To what degree did you have to push yourself to get things done because of your fatigue?”), physical function (example item: “Are you able to climb up five steps?”), and ability to participate in social roles and activities (example item: “I have trouble doing all of the activities with friends that are really important to me”) [25, 26]; and the COPD-SIB (example item: “It frustrated me that I couldn’t do everything I wanted to do anymore”) [16]. The PROMIS ability to participate in social roles and activities item bank was used in its entirety (35 items). We included a sub-set of the other two PROMIS item banks: we selected 50 fatigue and 63 physical function items. Item selection was performed by JP who has ample experience with COPD patients and COPD research, and reviewed by an international colleague of JP's with comparable experience. The COPD-SIB contains 46 items: both newly written items, and (adapted versions of) items from the SGRQ-C, the Quality of Life for Respiratory Illness Questionnaire (QoL-RIQ), the COPD Assessment Test, the Maugeri Respiratory Failure Questionnaire Reduced Form (MRF26), and the VQ11 [27–30]. In our application, a higher latent trait score indicated better HRQL for all domains.

### Test design

Multidimensional calibrations are not currently available for the PROMIS general population sample, and therefore the PROMIS calibrations cannot be used in the current study. In order to facilitate multidimensional calibration, our test design needed to be constructed in a way that would allow for item parameter estimation as well as estimation of the covariance structure among the domains. We used a booklet design, whereby the total number of items was distributed among three booklets each containing around 100 items. The booklets were linked using ten anchor items per domain (this type of linking is also known as *alternate form equating* or *common*-*item equating*). Each booklet contained items pertaining to at least two domains.

### Calibration sample

The following inclusion criteria were used: a medical diagnosis of COPD; sufficient oral and written mastery of the Dutch language; and being able to complete a questionnaire. HCPs (pulmonologists, general practitioners, physiotherapists, and nurse practitioners) were recruited by JP, through his professional network. HCPs distributed the questionnaires accompanied by an information letter among COPD patients attending their clinics from October 2014 through December 2015. Of the 1500 printed booklets, 795 were returned by the end of December 2015. Our sample had a mean age of 67.2 years (*SD* = 10.08), and consisted of 52.7% men. More detailed patient characteristics are reported in Supplement 1.

### Data preparation

All items in the item bank were scored on a 5-point Likert scale ranging from 0 to 4. In total, 10 different types of answer categories were used (depending on the domain and item formulation), for example, *without any difficulty, with a little difficulty, with some difficulty, with much difficulty, unable to do* or *never, rarely, sometimes, usually, always.* Twenty-eight percent of the items showed low endorsement (fewer than 10 responses) for one or more of its categories. Following Paap et al. [16], for 55 out of 194 items, item response categories that showed low endorsement (fewer than 10 responses) were merged with adjacent categories. Among these 55 items, 18 pertained to the fatigue domain, 23 to physical function, 2 to ability to participate in social roles and activities, and 12 to the COPD-SIB. For the majority of these items (51), the lowest two or highest two categories were collapsed. In the other cases, either the lowest or highest three categories were collapsed, or both the lowest two and the highest two. Note that items having different numbers of response categories due to merging does not constitute a problem for the IRT model used (multidimensional GRM).

### Multidimensional IRT calibration

The multidimensional graded response model was used to obtain item parameter estimates and estimates of the covariance structure.

The probability of a response in category *j* in item *i* with *m* total response categories, $P(X_{ij} = 1|\varvec{\theta})$, is given by

$$P_{ij} \left( \theta \right) = \left\{ {\begin{array}{*{20}c} {1 - \varPsi \left( {\varvec{\alpha}^{\varvec{'}}\varvec{\theta}- \beta_{i1} } \right)} & {{\text{if}}\quad j = 0,} \\ {\varPsi \left( {\varvec{\alpha}^{\varvec{'}}\varvec{\theta}- \beta_{ij} } \right) - \varPsi \left( {\varvec{\alpha}^{\varvec{'}}\varvec{\theta}- \beta_{{i\left( {j + 1} \right)}} } \right)} & {{\text{if}}\quad 0 < j < m,} \\ {\varPsi \left( {\varvec{\alpha}^{\varvec{'}}\varvec{\theta}- \beta_{im} } \right)} & {{\text{if}}\quad j = m,} \\ \end{array} } \right.$$

where Ψ(*x*) is the logistic function,

$$\varPsi \left( x \right) = \frac{\exp \left( x \right)}{1 + \exp \left( x \right)},$$

and $\varvec{\alpha}^{\varvec{'}}\varvec{\theta}$ denotes the dot product of the vector of discrimination parameters and latent traits. To ensure that the probabilities are always positive, response categories must be sorted by difficulty, $\beta_{{i\left( {j + 1} \right)}} > \beta_{ij}$ for 0 < *j* < *m*.

Up to five parameters were calculated for each item *i*: one discrimination parameter (denoted *α*_*i*_) and several *β*_*ij*_ parameters; the number of *β*_*ij*_ parameters equals the number of categories minus one. The *β*_*ij*_ parameter is related to the difficulty with which a respondent will reach the *j*th step of each item. Note that in unidimensional IRT, two types of parametrization can be used for *x*: $\alpha \left( {\theta - \beta } \right)$ or *αθ* − *β*. In multidimensional IRT, $\varvec{\alpha}^{\varvec{'}}$ is a vector containing an *α* value for each dimension; here, only the *αθ* − *β* parametrization can be used. Some software packages, such as IRTPRO, calculate “easiness” rather than “difficulty” parameters. In IRTPRO, this parameter is denoted as *c*. The *β*_*ij*_ parameter described above equals the negative value of the *c*-parameter. The estimates of the item parameters and covariance structure were obtained using the software package IRTPRO [31].

A multivariate normal distribution was assumed for the four latent traits, with variances fixed to 1 and the covariances being estimated freely. The estimated correlation matrix among the four domains Φ equalled

$$\left[ {\begin{array}{cccc} 1 & {0.77} & {0.87} & {0.77} \\ {0.77} & 1 & {0.84} & {0.76} \\ {0.87} & {0.84} & 1 & {0.77} \\ {0.77} & {0.76} & {0.77} & 1 \\ \end{array} } \right],$$

with rows and columns representing fatigue, physical function, ability to participate in social roles and activities, and the COPD-SIB, respectively. The item parameters are presented in Supplement 2. The discrimination parameters were relatively high for all domains (range: 0.82–5.40), which is quite common for clinical measures [32], and the *β*_*ij*_ parameters showed a good spread (range: −7.57 to 7.67). Measurement precision for *θ*-estimates was excellent (RMSE < 0.3 for all domains). The direction of bias was in line with the expected shrinkage (which is the result of the implementation of a Bayesian estimator): positive *θ*-values tended to be slightly underestimated and low negative *θ*-values tended to be overestimated. See Supplement 3 for RMSE and bias plots.

### Data generation and CAT simulations

CAT simulations were run with the package ShadowCAT [33] in R [34]. To evaluate the item usage rates of all individual items in our item bank, responses were generated based on 21000 vectors of pre-specified *θ*-values—1000 for every increment of 0.2 on the multidimensional *θ*-scale between values −2 and 2. The Maximum A Posteriori (MAP) estimator was used in all simulations to estimate *θ*, at all stages of the CAT. The covariance matrix Φ estimated using the multidimensional GRM was used as a prior. Following Segall [19], item selection was based on the value of the determinant of the posterior information matrix. Diao and Reckase [35] refer to this item selection method as *Bayesian Volume Decrease*, whereas Yao [36] simply abbreviates it as *Volume* or *Vm*. One random item per domain was administered at the start in order to obtain initial *θ*-values to initialize the CAT. The CAT was terminated, when the termination rule (threshold standard error of measurement *SE*(*θ*) < 0.316)^3^ was met for all four domains. Item selection for a particular dimension was terminated, when the *SE*-threshold had been met for that dimension.

### Outcome variables

The outcome variables in this study were overuse and active domain/bank size, all conditional on *θ*. Each of the outcome variables will be reported by domain as well as across domains (i.e., at item bank level). An item was considered overused when its usage rate was higher than the expected item usage rate,^4^ defined as the average test length for a given *θ*-value divided by the total bank size (194). Active domain/bank size was calculated as total domain or bank size minus items that were never used in the respective domain or overall bank.

## Results

**Fig. 1:**
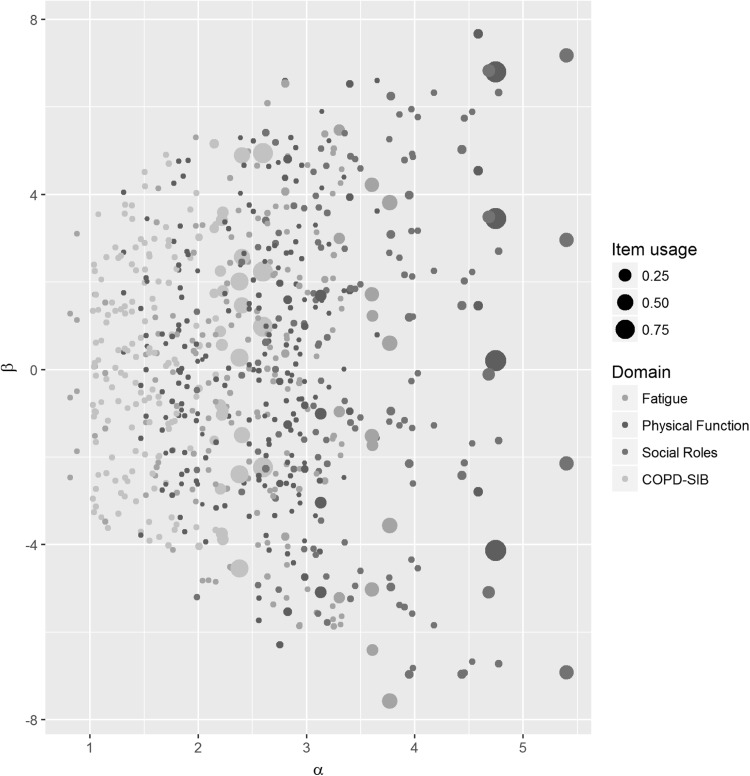
Scatterplot with discrimination values on the *x*-axis and *β*-parameter values (related to difficulty) on the *y*-axis. *Dots* represent item steps. The size of the *dots* increases as a function of item usage rate. See online for color version

The results of the CAT simulations are summarized in Tables 1 and 2, Fig. 1, and Supplement 4. Table 1 illustrates that there was—as could be expected—quite some diversity in active bank size across the different *θ*-values. For average *θ*-values, the overall active bank size was 9–10%; this number quickly increased as *θ*-values became more extreme. For values of −2 and +2, the overall active bank size increased fourfold to 39–40%! Unsurprisingly, CATs for more extreme *θ*-values (−2 and +2) were generally longer than for less extreme values (average length of 20.5 and 18.9 versus 14.1, 13.3, and 13.0 for *θ*-values; −1, 0, and +1, respectively). However, the active bank size increased at a steeper rate than the test length, for increasing absolute *θ*-values. There was also considerable diversity in active bank size across domains. For average *θ*-values, the active domain size for fatigue and physical function was 5–6%, compared to 9–11% for ability to participate in social roles and activities, and 17% for the COPD-SIB. For extreme *θ*-values, almost all ability to participate in social roles and activities items were used; this finding can be directly linked to the item parameter distributions for this bank (high discrimination parameters combined with broad coverage on the *θ*-scale); see Fig. 1.

**Table 1:** Active bank size (expressed in %) for *θ*-values ranging between −2 and +2

*θ*	Total bank	Fatigue	Physical function	Social roles	COPD-SIB
−2	40	26	33	71	39
−1.8	24	20	16	37	30
−1.6	24	20	17	34	28
−1.4	19	14	16	23	26
−1.2	17	10	13	23	26
−1	15	10	14	20	20
−0.8	13	8	13	14	20
−0.6	10	8	8	11	15
−0.4	9	6	6	11	15
−0.2	9	6	6	11	15
0	9	6	5	9	17
0.2	9	6	5	11	17
0.4	9	6	5	9	17
0.6	10	8	5	11	17
0.8	9	10	3	9	17
1	10	12	3	11	17
1.2	15	18	6	20	22
1.4	17	20	6	23	24
1.6	19	22	10	20	26
1.8	24	26	14	31	30
2	39	30	16	97	37
Full bank size^a^	194	50	63	35	46

^a^Number of available calibrated items in each domain/the total bank

**Table 2:** Overused items (expressed in %) for *θ*-values ranging between −2 and +2

*θ*	Total bank	Fatigue	Physical function	Social roles	COPD-SIB
−2	15	10	13	17	22
−1.8	14	8	13	17	20
−1.6	12	6	11	17	17
−1.4	11	6	8	17	17
−1.2	9	6	8	9	15
−1	8	6	6	6	15
−0.8	8	6	6	6	13
−0.6	7	6	5	6	13
−0.4	7	6	5	6	11
−0.2	7	6	5	6	11
0	7	6	5	6	11
0.2	7	6	3	9	13
0.4	8	6	3	9	15
0.6	7	6	3	6	15
0.8	7	6	3	6	15
1	8	8	3	6	17
1.2	9	10	3	6	17
1.4	10	12	3	9	17
1.6	11	12	6	11	17
1.8	13	12	8	20	15
2	14	14	8	17	20

Overused items are defined as items whose usage rate exceeded the expected usage rate (average test length for a given *θ*-value divided by the total bank size)

Comparing Table 2 (percentage of overused items) to Table 1 (active bank size) shows that—for the total bank and average *θ*-values—overused items dominated the active part of the multidimensional item bank; there was 78% overlap between overused items and active bank size. For more extreme *θ*-values, the overused items made up a much smaller part of the active bank size: here the overlap was only 35%.

Figures 1–4 in Supplement 4 illustrate that there are 12 items that have relatively high item usage rates over a wide range of *θ*-values: FATIMP1 (“To what degree did you have to push yourself to get things done because of your fatigue?”), FAMTIMP9 (“How often did your fatigue make it difficult to plan activities ahead of time?”), FATIMP29 (“How often were you too tired to leave the house?”), PFB1 (“Are you able to climb up five steps?”), PFB44 (“Does your health now limit you in doing moderate activities, such as moving a table, pushing a vacuum cleaner, bowling, or playing golf?”), SRPPER20 (“I have trouble doing all of the activities with friends that are really important to me”), SRPPER23 (“I have trouble doing all of my usual work (include work at home)”), SGRQ12 (“Please, indicate whether the following activity causes shortness of breath. If the weather influences your complaints, assume the weather conditions are favorable, when you answer this question. Getting washed or dressed”), SGRQ13 (“Please, indicate whether the following activity causes shortness of breath. If the weather influences your complaints, assume the weather conditions are favorable, when you answer this question. Walking around the home.”), SGRQ26 (“I get afraid or panic when I cannot get my breath.”), SGRQ42R1a (“My breathing problems make it difficult to do light gardening, such as weeding.”), and SGRQ42R1b (“My breathing problems make it difficult to do things such as dancing, playing golf, or playing bowls.”).

Some items, such as CSIB13 (“It frustrated me that I couldn’t do everything I wanted to do anymore”) and SGRQ31 (“Everything seems too much of an effort.”), show two peaks; something typical for polytomous data. Polytomous items have more than one *β* parameter and thus cover a wider *θ*-range. A polytomous item can have more than one peak in its item information function, which would translate into more than one peak in the item usage plot. Longer CATs are needed to obtain reliable estimates of very low or high *θ*-values, which explains why as many as 38 items show relatively high item usage rates for low or high *θ*-values only.

In Fig. 1, the item step parameters are plotted against the discrimination parameters for each domain. The figure clearly shows that within each domain, the items with the highest discrimination values had the highest item usage rates. These items typically covered a wide range of *θ*-values.

## Discussion

In this study, we evaluated active bank size and item overuse/overexposure in a recently developed MCAT designed to measure HRQL in COPD patients using four correlated domains. Three generic PROMIS domains were used: the PROMIS domains fatigue, physical function, and ability to participate in social roles and activities [25, 26]; as well as a COPD-specific item bank (the COPD-SIB) which was recently developed [16]. We found that, for average latent trait values, the overall active bank size was 9–10%; compared to 39–40% for more extreme latent trait values (−2 and +2). Furthermore, as expected, domains with highly discriminating items were overrepresented in the active part of the multidimensional bank. For average latent trait values, the active part of the bank was almost entirely populated by overused items. In contrast, for more extreme latent trait values, the active part of the multidimensional bank was dominated by underused items. The number of items that showed good item usage and covered almost the entire latent trait range varied between 2 (physical function) and 5 (COPD-SIB) per domain.

We used a multidimensional item bank consisting of 194 items (35–63 items per domain). Given that we developed a MCAT without content constraints and with no exposure control, our results indicate that the MCAT was working as intended: for average latent trait values, a small number of highly discriminating items was selected; for more extreme values, the item bank usage was more balanced. However, our results also showed that a relatively large part of the multidimensional item bank was never used (60%). The active part of the bank consisted of 77 items at most, across the four domains. This may indicate that—if these findings can be generalized—roughly 19 polytomous items per domain might suffice, when developing a multidimensional bank populated by items with high discrimination parameters that adequately cover the latent trait range of interest, and with high correlations among domains. Research focusing on unidimensional CATs has shown that CATs based on polytomous rather than dichotomous items can be performed with substantially smaller item banks; an item bank of 30 items may be sufficient for polytomously scored health outcomes [38, 39]. Our results suggest that MCAT potentially requires smaller item banks than UCAT. It would be interesting to study this further in a future study.

Item usage has received little attention in the field of clinical (psychological/health) measurement so far. One exception concerns developing IRT/CAT-based short forms. Several authors have suggested that CAT simulations can be used to select the most appropriate items for inclusion in a short form [40–42]. In these studies, typically the entire item pool is administered, after which the rank order in which the items were administered is calculated and averaged over all simulees. The “best” items (items with the lowest average CAT presentation ranks) would then be selected for the short form [41]. Items for the newest PROMIS short forms were selected based on the maximum interval information and CAT simulations (highest average administration rank) [43], making their measures easily accessible in situations where CAT may not be feasible. Because static short forms will be typically targeted at a relatively wide latent trait range, they are relatively long compared to CATs, especially for respondents with average latent trait values. Furthermore, although a short form may achieve adequate measurement precision for average to moderately high latent trait scores, CATs provide much better precision at the extremes [41, 42]. Our results showed how active bank size and the rate of overused items also depended on latent trait values. In other words, which items are the “best” items (in terms of administration rank/usage) depends largely on the respondent’s latent trait values. This is not something that can be satisfactorily addressed in a short form.

Another topic which has received little attention in our field is the influence of capitalization on item calibration error. Since the item selection criterion most frequently used is a direct function of the discrimination parameter, item selection is sensitive to large standard errors of discrimination parameters [44, 45]. Typically, extreme discrimination parameter estimates tend to be associated with larger standard errors [46]. Furthermore, the smaller the selection ratio (CAT length divided by total item bank), the larger the danger of capitalization on chance [47]. Capitalization on item calibration error may lead to overestimation of test information and underestimation of the standard errors of latent trait estimates [46]. In this light, having a small set of items with very high item usage rates (and a large set not being used at all) may be worrying, regardless of the issue of test security. In this study, we did find a strong correlation (0.82) between estimated discrimination parameters and their respective standard errors. However, penalizing items with the highest discrimination parameter estimates (for example, by increasing the estimates by 1 or 2 times their corresponding standard error), would have had a very insubstantial effect on their ranking (data not shown). This being said, if we would have penalized items with relatively high standard errors during the CATs, test length would most likely have been somewhat longer, and subsequently the active size of the item bank would also have been larger. Since estimates are typically (also in our case; data not shown) more precise when using a multidimensional rather than unidimensional IRT models to calibrate the items, the impact of item calibration error can be expected to be smaller than if we had used separate unidimensional CATs. Research investigating the potential protective effect of multidimensional IRT and CAT on the consequences of capitalization on item calibration error is needed.

## Conclusion

With this study, we extended the literature on item usage rates to multidimensional health measurement. We showed what happens when realistic CAT settings (typical for health measurement) are used: a relatively small number of highly discriminating items is selected. Currently, PROMIS item banks differ widely in length. Our results strengthen the claim that relatively short item banks may suffice when using polytomous items (and no content constraints/exposure control mechanisms), especially when using MCAT. This may be particularly relevant to item bank developers. However, if researchers or clinicians want to be able to influence the content (to ensure validity), different item selection procedures are necessary; in such instances, a larger item bank will be needed.

## Electronic supplementary material

Below is the link to the electronic supplementary material.

Supplementary material 1 (PDF 141 kb)

Supplementary material 2 (PDF 371 kb)

Supplementary material 3 (PDF 148 kb)

Supplementary material 4 (PDF 638 kb)

## Footnotes

1 In a unidimensional setting, Fisher information, which varies as a function of the latent trait value, is often used. When the item location parameter equals the latent trait value, Fisher information increases monotonically as the value of the discrimination parameter increases; therefore, the item selection rule based on Fisher information will select an item with a location close to the current latent trait estimate and a discrimination that is as large as possible (see, e.g., [18]). In MCAT, item information is no longer expressed by a single value; instead, item selection typically depends on the value of the determinant of the posterior information matrix (this value is computed and evaluated for each of the remaining items in the multidimensional item bank, and the item for which the value is largest is selected) [19].

2 In interviews with healthcare professionals [24], the target group that was to use our CAT, the majority indicated that they were not interested in a global score, but instead favored separate scores for each dimension (data not shown).

3 In unidimensional models, an *SE*-value of 0.316 corresponds to a local reliability of 0.90 when a variance of 1 for *θ* is assumed (see, e.g., [37]).

4 Note that *usage* was operationalized as selection in the adaptive part of the CAT (items selected at random to be used as start items to initialize the CAT were ignored in calculating the outcome variables).
