# Supplementary material for: Item usage in a multidimensional computerized adaptive test (MCAT) measuring health-related quality of life
Source: Qual Life Res. 2017 Jun 23;26(11):2909–18. doi: 10.1007/s11136-017-1624-3 (PMC5655597; doi:10.1007/s11136-017-1624-3)
Supplement: Supplementary file 1 — Supplementary material 1 (PDF 141 kb) [file 11136_2017_1624_MOESM1_ESM.pdf]

Supplement 1 Patient characteristics

	N = 795
**Age**	
Mean (SD)	67.2 (10.08)
Missings (%)	6.0
**Gender (%)**	
Male	52.7
Female	45.7
Missings	1.6
**Marital status (%)**	
Not married/not cohabiting	9.2
Not married/cohabiting	5.3
Married	60.4
Widow/widower	13.6
Divorced	9.2
Missings	2.4
**Highest educational attainment (%)**	
Primary school	15.2
Prevocational education	32.3
Preparatory secondary vocational education/junior general secondary education	17.5
Secondary vocational education	13.7
Senior general secondary school/pre-university education	5.2
Higher professional education	10.7
University education	3.1
Missings	2.3
**Current situation (%)**	
Full-time employment	8.4
Part-time employment	6.5
Homemaker	9.6
Unemployed	3.3
Disabled	13.7

Retired	56.4
Missings	2.1
**Children (%)**	
No	15.0
Only children living away from home	72.2
One or more children living at home	10.8
Missings	2.0
**Mother tongue (%)**	
Dutch	97.0
Moroccan/Turkish/Arabic	.6
German/French/English	.9
Other	.4
Missings	1.1
**Sports (frequency) (%)**	
I do not participate in sports (anymore)	45.2
A few times a month	1.8
1-2 times a week	30.2
Appr. 2-3 times a week	16.7
More than 3 times a week	4.2
Missings	2.0
**Cigarettes (per day) (%)**	
None	75.3
Less than 5 cigarettes	5.5
5-10 cigarettes	7.0
11-20 cigarettes	7.2
21-30 cigarettes	2.5
31- 40 cigarettes	.3
More than 40 cigarettes	.1
Missings	2.0

This online supplement accompanies the following paper: *Item usage in a multidimensional computerized adaptive test measuring health-related quality of life* written by Muirne C. S. Paap, Karel Kroeze, Caroline B. Terwee, Job van der Palen, and Bernard P. Veldkamp.

Contact details: Muirne Paap, m.c.s.paap@cemo.uio.no
