# Supplementary material for: Item usage in a multidimensional computerized adaptive test (MCAT) measuring health-related quality of life
Source: Qual Life Res. 2017 Jun 23;26(11):2909–18. doi: 10.1007/s11136-017-1624-3 (PMC5655597; doi:10.1007/s11136-017-1624-3)
Supplement: Supplementary file 2 — Supplementary material 2 (PDF 371 kb) [file 11136_2017_1624_MOESM2_ESM.pdf]

Supplement 2 Item parameters for the multidimensional graded response model

Item code	a	SE(a)	b1	SE(b1)	b2	SE(b2)	b3	SE(b3)	b4	SE(b4)
Fatigue										
AN1	2.71	0.17	-2.74	0.19	-0.12	0.13	2.20	0.17		
AN2	2.81	0.25	-5.21	0.45	-2.16	0.25	0.78	0.20	3.72	0.34
AN5	1.04	0.14	-1.51	0.17	0.07	0.14	1.72	0.18		
AN7	0.88	0.13	-1.86	0.18	-0.49	0.13	1.13	0.15	3.11	0.27
AN8	1.14	0.10	-3.47	0.23	-1.35	0.12	-0.08	0.10	1.53	0.12
AN12	2.09	0.23	-4.81	0.45	-1.86	0.21	-0.31	0.16		
AN14	1.94	0.19	-2.74	0.24	-0.94	0.17	0.66	0.16		
AN15	2.64	0.16	-3.96	0.24	-1.02	0.14	0.82	0.13	3.34	0.21
AN16	2.93	0.27	-5.86	0.51	-2.73	0.27	-0.30	0.18	1.92	0.23
FATEXP2	2.30	0.21	-4.51	0.39	-0.53	0.18	2.44	0.25	4.38	0.37
FATEXP6	2.04	0.14	-4.82	0.32	-1.35	0.13	0.79	0.12	3.30	0.20
FATEXP7	2.31	0.19	-4.54	0.36	-1.21	0.18	1.01	0.18	3.03	0.25
FATEXP18	2.64	0.22	-4.73	0.39	0.02	0.17	3.58	0.31	6.09	0.51
FATEXP20	1.97	0.18	-3.01	0.27	0.37	0.17	3.29	0.28	5.31	0.45
FATEXP22	2.80	0.23	-3.82	0.32	0.36	0.18	4.06	0.34	6.54	0.55
FATEXP24	1.61	0.17	-2.23	0.22	0.38	0.16	3.36	0.30		
FATEXP26	2.66	0.24	-2.76	0.28	0.51	0.19	2.47	0.25		
FATEXP28	1.96	0.19	-1.93	0.21	0.43	0.17	2.67	0.25		
FATEXP38	2.46	0.20	-2.49	0.23	0.68	0.18	2.46	0.23	4.99	0.39
FATEXP45	2.71	0.24	-2.38	0.25	0.26	0.19	3.73	0.32		
FATEXP46	2.09	0.19	-2.29	0.21	-1.11	0.17	0.56	0.16	1.11	0.17
FATEXP49	2.37	0.21	-4.48	0.39	-1.00	0.19	1.24	0.19	3.64	0.31
FATEXP54	1.63	0.17	-2.12	0.22	0.82	0.17	4.14	0.36		
FATEXP56	3.17	0.30	-2.54	0.29	2.07	0.27	5.38	0.50		
FATIMP1	3.61	0.29	-6.40	0.56	-1.72	0.25	1.23	0.23	4.24	0.36
FATIMP2	1.76	0.16	-4.12	0.36	-1.84	0.19	-0.06	0.15	1.23	0.17
FATIMP4	3.32	0.22	-5.63	0.35	-1.70	0.17	1.39	0.17	4.58	0.29
FATIMP5	3.30	0.27	-5.22	0.43	-0.96	0.21	3.00	0.30	5.47	0.45
FATIMP8	1.51	0.18	-2.87	0.26	-1.20	0.17	0.35	0.16		
FATIMP9	3.60	0.30	-5.02	0.44	-1.52	0.25	1.72	0.25	4.22	0.37
FATIMP10	3.24	0.27	-5.70	0.49	-1.53	0.23	2.09	0.25	4.95	0.43
FATIMP11	1.60	0.16	-2.42	0.22	-0.23	0.14	1.20	0.16		
FATIMP13	2.82	0.22	-4.46	0.35	-1.91	0.22	0.71	0.18	3.15	0.28
FATIMP15	2.82	0.23	-4.04	0.35	-0.44	0.19	2.29	0.25	4.88	0.39
FATIMP16	3.31	0.29	-5.82	0.48	-1.95	0.23	1.63	0.23	4.26	0.38
FATIMP19	3.01	0.20	-5.02	0.31	-0.91	0.15	1.77	0.17	4.68	0.29
FATIMP21	2.69	0.23	-5.37	0.47	-2.89	0.28	-0.27	0.19	2.16	0.23

FATIMP22	2.47	0.25	-2.10	0.24	0.02	0.18	3.08	0.30		
FATIMP25	2.27	0.19	-2.72	0.24	-0.19	0.16	1.97	0.21		
FATIMP26	3.09	0.24	-5.55	0.44	-2.37	0.24	0.30	0.18	3.14	0.28
FATIMP29	3.77	0.25	-7.57	0.54	-3.56	0.27	0.61	0.16	3.82	0.26
FATIMP38	2.16	0.19	-4.85	0.42	-2.61	0.24	-0.73	0.16	0.80	0.17
FATIMP40	0.82	0.12	-2.46	0.21	-0.64	0.13	1.28	0.15		
FATIMP42	3.25	0.26	-5.87	0.49	-1.51	0.22	2.62	0.27	5.06	0.41
FATIMP47	2.93	0.18	-5.83	0.37	-2.27	0.18	-0.24	0.14	2.10	0.17
FATIMP49	3.24	0.20	-5.41	0.33	-1.58	0.16	0.90	0.15	4.03	0.25
FATIMP52	2.26	0.22	-2.73	0.26	-0.85	0.18	1.56	0.20		
FATIMP53	2.55	0.15	-4.00	0.24	-1.08	0.14	1.18	0.14	3.46	0.21
HI12	1.80	0.18	-2.81	0.26	-0.84	0.17	0.80	0.17		
HI7	2.54	0.21	-3.28	0.28	-0.98	0.18	1.34	0.19	4.66	0.37
Physical Function										
PFA1	1.91	0.17	-0.17	0.15	2.63	0.24	4.78	0.39		
PFA3	1.83	0.12	-3.38	0.20	-0.06	0.11	1.87	0.14	3.28	0.19
PFA4	2.96	0.27	-0.67	0.20	2.53	0.28	4.67	0.40		
PFA5	2.42	0.19	-2.59	0.23	0.32	0.17	2.65	0.24	4.67	0.35
PFA6	2.85	0.27	-2.43	0.25	-0.26	0.18	1.41	0.21		
PFA10	2.28	0.21	-1.06	0.18	0.03	0.16	1.34	0.19	3.22	0.28
PFA11	3.34	0.19	-2.46	0.17	0.01	0.14	1.80	0.17	4.40	0.26
PFA13	1.82	0.16	-0.32	0.15	0.90	0.16	2.27	0.21	4.76	0.38
PFA15	2.16	0.22	-3.16	0.28	-1.66	0.20	-0.22	0.16		
PFA17	2.47	0.22	-3.36	0.29	-1.15	0.19	0.10	0.18	1.51	0.20
PFA19R1	2.80	0.39	4.38	0.56	6.59	0.77				
PFA21	2.85	0.17	-1.38	0.14	0.87	0.14	2.68	0.18	5.30	0.31
PFA23	2.82	0.16	-2.60	0.17	-0.72	0.13	0.56	0.13	2.66	0.18
PFA25	3.65	0.30	-1.56	0.23	0.79	0.21	3.62	0.35	6.60	0.57
PFA31R1	1.99	0.18	-2.62	0.24	-1.01	0.18	0.20	0.16	2.26	0.22
PFA33	1.53	0.15	0.54	0.15	2.02	0.20	3.45	0.28		
PFA37	2.04	0.21	-3.08	0.28	-1.48	0.19	0.23	0.16		
PFA39R1	1.78	0.30	2.93	0.36	4.26	0.47				
PFA41	1.90	0.16	-2.31	0.20	-0.39	0.15	1.11	0.17	2.70	0.23
PFA42	2.90	0.23	-1.16	0.19	0.50	0.18	1.95	0.22	4.31	0.35
PFA45	1.84	0.25	-2.61	0.26	-0.92	0.18				
PFA47	2.82	0.27	-4.21	0.36	-2.12	0.23	-0.08	0.18		
PFA52	1.88	0.20	-3.71	0.33	-1.90	0.20	-1.06	0.17	0.53	0.16
PFA53	3.13	0.27	-5.09	0.43	-3.03	0.29	-1.01	0.21	1.69	0.23
PFA55	2.94	0.27	-3.05	0.28	-1.47	0.21	0.36	0.18		
PFA56	2.07	0.20	-3.94	0.34	-2.02	0.21	0.31	0.16		
PFB1	4.75	0.41	-4.13	0.41	0.20	0.24	3.45	0.38	6.80	0.61
PFB3	3.08	0.26	-3.34	0.30	-0.63	0.19	1.43	0.22	3.30	0.30

PFB5R1	2.74	0.24	-0.25	0.18	2.32	0.25	4.73	0.40		
PFB7	2.90	0.26	0.13	0.19	2.92	0.30	5.07	0.45		
PFB8R1	3.21	0.20	-0.39	0.13	1.29	0.15	2.87	0.21	5.23	0.32
PFB10	2.98	0.26	-4.74	0.41	-2.28	0.25	-0.82	0.20	0.99	0.20
PFB11	2.86	0.29	-3.24	0.30	-1.96	0.24	-0.13	0.19		
PFB12	2.93	0.23	-2.86	0.25	-0.67	0.18	0.68	0.18	3.28	0.29
PFB13	2.98	0.26	-3.91	0.34	-2.13	0.24	-0.27	0.19	1.60	0.23
PFB17	1.98	0.19	-2.55	0.23	-1.17	0.17	0.58	0.16		
PFB24	2.51	0.22	0.00	0.17	2.05	0.23	3.61	0.32	5.23	0.44
PFB26	3.12	0.38	-4.16	0.43	-2.54	0.30	-1.02	0.23		
PFB28R1	1.52	0.15	-1.06	0.15	0.00	0.14	0.77	0.15	2.09	0.20
PFB32	1.76	0.17	-2.86	0.24	-1.85	0.19	-0.73	0.16	0.76	0.16
PFB36	2.88	0.22	-4.42	0.29	-2.65	0.20	-1.02	0.15		
PFB39R1	1.85	0.18	-2.40	0.22	-0.78	0.16	0.02	0.15	1.65	0.19
PFB42	1.82	0.16	-1.05	0.16	-0.02	0.15	1.01	0.17	2.38	0.21
PFB43	2.56	0.16	-5.73	0.38	-2.42	0.17	-0.02	0.12	1.70	0.15
PFB44	4.58	0.43	-2.79	0.31	1.46	0.29	4.55	0.50	7.67	0.76
PFB48	2.56	0.21	-5.23	0.44	-2.52	0.23	-0.25	0.17	1.40	0.19
PFB49	2.71	0.21	-3.34	0.28	-1.14	0.19	0.99	0.19	2.83	0.26
PFB50	2.82	0.22	-5.53	0.47	-1.26	0.20	1.60	0.21	4.81	0.39
PFB54	2.75	0.16	-6.28	0.43	-2.60	0.17	-0.27	0.13	1.98	0.16
PFB56R1	1.67	0.18	-2.75	0.24	-1.38	0.17	-0.51	0.15	0.81	0.16
PFC6R1	2.63	0.23	-3.79	0.31	-1.96	0.22	-0.79	0.18	0.99	0.18
PFC7R1	1.86	0.36	4.42	0.61						
PFC10	2.47	0.21	-1.70	0.20	1.39	0.20	3.76	0.31		
PFC12	3.40	0.29	-0.95	0.21	1.80	0.24	3.93	0.36	6.53	0.56
PFC13R1	2.39	0.26	2.40	0.29	4.03	0.39	5.30	0.49		
PFC29	1.70	0.18	-2.61	0.24	-1.91	0.20	-1.09	0.17	0.60	0.16
PFC32	1.31	0.14	0.40	0.15	1.75	0.19	2.68	0.23	4.05	0.34
PFC35	3.14	0.32	1.58	0.25	4.15	0.44	5.90	0.58		
PFC36R1	2.65	0.21	-1.61	0.20	0.64	0.18	2.76	0.26	4.64	0.36
PFC37	2.25	0.18	-3.11	0.25	-0.37	0.16	1.74	0.20	3.37	0.27
PFC38	2.61	0.15	-2.65	0.17	-1.06	0.13	0.35	0.12	2.47	0.17
PFC41	1.47	0.14	-3.79	0.33	-1.57	0.17	-0.37	0.14	1.40	0.17
PFC56	2.65	0.18	-4.13	0.25	-1.71	0.15	0.79	0.13		
*Social roles**										
RP1	3.35	0.20	-3.94	0.24	-0.72	0.14	2.76	0.20	5.40	0.33
RP6	2.88	0.24	-2.28	0.25	1.06	0.19	3.74	0.32		
SRPPER_C	3.19	0.26	-5.78	0.50	-2.16	0.24	0.74	0.19	3.58	0.31
SRPPER01	2.74	0.23	-5.03	0.43	-1.48	0.21	1.17	0.21	3.13	0.29
SRPPER02	3.13	0.18	-3.45	0.22	-0.26	0.13	2.79	0.20	5.05	0.30
SRPPER03	3.50	0.20	-4.60	0.28	-1.10	0.15	1.95	0.17	4.60	0.28

SRPPER04	2.71	0.16	-2.94	0.19	0.00	0.13	2.89	0.20	5.19	0.30
SRPPER05	3.90	0.32	-5.43	0.46	-1.16	0.23	2.17	0.28	4.79	0.43
SRPPER06	3.76	0.32	-4.75	0.42	-1.18	0.23	2.67	0.29	5.27	0.46
SRPPER07	3.97	0.33	-5.57	0.47	-1.33	0.24	2.13	0.28	4.93	0.44
SRPPER08	3.98	0.33	-6.82	0.59	-2.60	0.29	1.22	0.22	4.86	0.42
SRPPER09	4.02	0.34	-4.53	0.41	-0.09	0.23	3.18	0.35	5.77	0.52
SRPPER11	4.18	0.36	-5.84	0.52	-1.27	0.24	2.26	0.28	6.33	0.57
SRPPER13	3.06	0.25	-4.09	0.35	-0.83	0.20	1.75	0.23	4.52	0.38
SRPPER14	4.44	0.26	-6.96	0.43	-2.42	0.21	1.46	0.18	5.03	0.32
SRPPER15	4.45	0.26	-6.93	0.43	-2.13	0.20	2.03	0.20	5.74	0.36
SRPPER16	2.62	0.22	-2.27	0.24	0.62	0.18	3.41	0.30	5.42	0.46
SRPPER17	3.11	0.26	-5.57	0.48	-2.00	0.24	0.56	0.20	2.91	0.29
SRPPER18	4.53	0.27	-6.67	0.41	-1.68	0.19	2.23	0.20	5.89	0.37
SRPPER20	5.40	0.46	-6.92	0.64	-2.15	0.30	2.96	0.33	7.18	0.65
SRPPER21	3.78	0.31	-4.97	0.44	-0.94	0.22	3.09	0.31	6.25	0.54
SRPPER22	3.11	0.26	-2.32	0.25	0.60	0.19	3.71	0.33		
SRPPER23	4.68	0.40	-5.09	0.46	-0.11	0.24	3.49	0.38	6.83	0.62
SRPPER26	3.86	0.33	-5.38	0.49	-1.28	0.23	2.56	0.29	5.83	0.51
SRPPER28	4.77	0.41	-6.72	0.59	-1.62	0.27	2.71	0.34	6.33	0.57
SRPPER31	2.40	0.21	-2.80	0.26	-0.14	0.17	2.46	0.24		
SRPPER35	3.18	0.26	-4.73	0.41	-1.61	0.22	1.81	0.23	4.66	0.39
SRPPER36	3.45	0.28	-4.94	0.43	-1.34	0.22	1.85	0.24	4.81	0.41
SRPPER37	3.97	0.33	-4.34	0.39	-0.26	0.22	3.17	0.34	5.95	0.52
SRPPER42	3.94	0.23	-6.97	0.44	-2.14	0.19	1.20	0.16	3.99	0.26
SRPPER43	1.98	0.13	-5.19	0.37	-2.29	0.16	-0.46	0.12	1.32	0.13
SRPPER46	3.41	0.20	-5.24	0.32	-1.21	0.15	1.85	0.17	4.87	0.29
SRPPER47	2.98	0.24	-4.10	0.36	-1.59	0.22	1.06	0.20	3.69	0.32
SRPPER54	3.08	0.25	-4.23	0.35	-1.43	0.21	1.59	0.23	3.97	0.35
SRPPER55	2.55	0.21	-4.92	0.43	-1.87	0.22	0.16	0.17	2.80	0.26
COPD-SIB										
QRIQ_E1R1	1.06	0.15	-2.73	0.27	-0.97	0.16	0.61	0.16		
QRIQ_E2	1.10	0.08	-3.37	0.19	-0.74	0.09	0.66	0.09	2.06	0.12
QRIQ_E3	1.23	0.14	-2.54	0.24	0.04	0.15	1.34	0.17	2.79	0.25
QRIQ_E4	1.29	0.14	-2.82	0.27	-0.07	0.15	1.38	0.18	2.61	0.24
CSIB1	1.15	0.10	-2.37	0.16	-0.22	0.10	1.07	0.12	2.57	0.17
VQ11_6R1	1.73	0.10	-1.35	0.10	1.98	0.12	2.95	0.15	4.71	0.24
CSIB2	1.55	0.16	-3.91	0.37	-1.20	0.17	0.48	0.15	2.59	0.23
CSIB3	1.08	0.08	-2.40	0.13	0.22	0.08	1.36	0.09	3.55	0.18
CSIB4	1.39	0.15	-1.32	0.17	1.33	0.17	2.41	0.21	3.75	0.32
CSIB5	1.06	0.12	-3.13	0.27	-1.66	0.17	-0.14	0.13	1.71	0.17
CSIB6	1.32	0.16	-2.48	0.23	-0.13	0.15	1.28	0.17	3.77	0.35
CSIB7	1.58	0.17	-2.38	0.23	-0.46	0.16	1.62	0.19		

CSIB8	1.04	0.14	-0.95	0.15	0.15	0.14	2.25	0.21		
CSIB9	2.14	0.19	-1.62	0.19	1.74	0.20	3.23	0.28	5.17	0.43
CSIB10	1.32	0.16	-2.90	0.27	0.48	0.15	1.44	0.18	3.56	0.32
CSIB11	1.78	0.10	-3.67	0.19	-1.18	0.10	0.23	0.09	2.57	0.13
CSIB12	1.51	0.15	-1.94	0.20	1.10	0.17	2.28	0.21	4.19	0.36
QRIQ_C8R1	2.01	0.20	-4.04	0.37	-0.43	0.17	0.54	0.17	3.02	0.28
MRF11R1	1.54	0.15	-3.48	0.29	-0.65	0.15	0.38	0.14	2.64	0.23
MRF18R1	1.80	0.10	-3.54	0.18	-1.00	0.09	0.14	0.09	2.30	0.13
MRF19R1	1.42	0.14	-3.01	0.25	-0.79	0.15	0.29	0.14	2.27	0.20
MRF20R1	1.63	0.19	-2.00	0.21	-0.62	0.16	2.01	0.21		
CSIB13	2.22	0.18	-3.88	0.31	-0.94	0.17	1.80	0.20	3.60	0.29
CSIB14	1.64	0.20	-3.83	0.37	-1.98	0.22	-0.77	0.17		
CSIB15	1.34	0.16	-2.55	0.22	-0.88	0.15	0.44	0.14		
CSIB16	1.75	0.20	-3.38	0.31	-1.21	0.18	0.80	0.17		
CSIB17	1.03	0.13	-2.95	0.25	-0.81	0.14	1.34	0.16		
CSIB18	1.16	0.14	-3.62	0.35	-1.17	0.16	1.25	0.17		
SGRQ3R1	1.70	0.16	-2.59	0.25	0.08	0.16	3.25	0.29	4.91	0.44
SGRQ12	2.20	0.20	-2.73	0.24	-0.81	0.17	0.87	0.17	2.25	0.22
SGRQ13	2.38	0.20	-4.54	0.37	-2.40	0.23	0.27	0.17	2.01	0.21
SGRQ18	1.20	0.14	-2.01	0.20	-0.40	0.14	1.53	0.17		
SGRQ19	1.60	0.16	-2.72	0.26	0.38	0.15	1.41	0.18	2.98	0.26
SGRQ20R1	1.47	0.16	-3.70	0.35	-0.53	0.15	0.73	0.16	2.98	0.27
SGRQ21R1	1.39	0.15	-2.32	0.21	0.58	0.15	1.55	0.17	3.44	0.29
SGRQ22	1.26	0.08	-3.30	0.17	-0.72	0.08	0.44	0.08	2.55	0.13
SGRQ23R1	1.93	0.19	-1.80	0.20	2.55	0.25	3.66	0.31		
SGRQ24R1	1.04	0.08	-3.26	0.17	-1.08	0.09	0.06	0.08	2.40	0.12
SGRQ25R1	1.72	0.18	-4.01	0.38	-1.24	0.17	0.15	0.15	2.83	0.26
SGRQ26	2.22	0.20	-3.75	0.33	-1.03	0.18	0.56	0.17	3.42	0.31
SGRQ27R1	1.42	0.15	-1.87	0.19	-0.30	0.15	2.79	0.25		
SGRQ29R1	2.16	0.21	-3.66	0.33	-0.11	0.17	1.17	0.19	3.32	0.30
SGRQ31	1.98	0.17	-3.61	0.29	-0.62	0.16	1.00	0.17	4.03	0.33
SGRQ42R1a	2.59	0.25	-2.23	0.24	0.98	0.19	2.24	0.24	4.95	0.44
SGRQ42R1b	2.40	0.23	-1.49	0.20	1.47	0.21	2.57	0.26	4.90	0.45
SGRQ43R1	1.50	0.16	-0.70	0.15	1.98	0.20	2.89	0.25	4.51	0.40

* Ability to participate in social roles and activities

Note that all items in the model were assigned to (loaded on) one domain each. Thus, the a’s printed here equal the estimated discrimination parameters for the domain in question while the other three parameters were constrained to 0. In this table, we collapsed the four original discrimination parameter estimate columns to improve readability.

This online supplement accompanies the following paper: *Item usage in a multidimensional computerized adaptive test measuring health-related quality of life* written by Muirne C. S. Paap, Karel Kroeze, Caroline B. Terwee, Job van der Palen, and Bernard P. Veldkamp.

If you want to use the COBD-SIB items and map the item parameters reported here to the item numbers (and item content) printed in the paper "The COPD-SIB: a newly developed disease-specific item bank to measure health-related quality of life in patients with chronic obstructive pulmonary disease", please contact the first author.

Contact details: Muirne Paap, m.c.s.paap@cemo.uio.no
