# Supplementary material for: Item usage in a multidimensional computerized adaptive test (MCAT) measuring health-related quality of life
Source: Qual Life Res. 2017 Jun 23;26(11):2909–18. doi: 10.1007/s11136-017-1624-3 (PMC5655597; doi:10.1007/s11136-017-1624-3)
Supplement: Supplementary file 3 — Supplementary material 3 (PDF 148 kb) [file 11136_2017_1624_MOESM3_ESM.pdf]

Supplement 3 Accuracy and precision of latent trait estimation

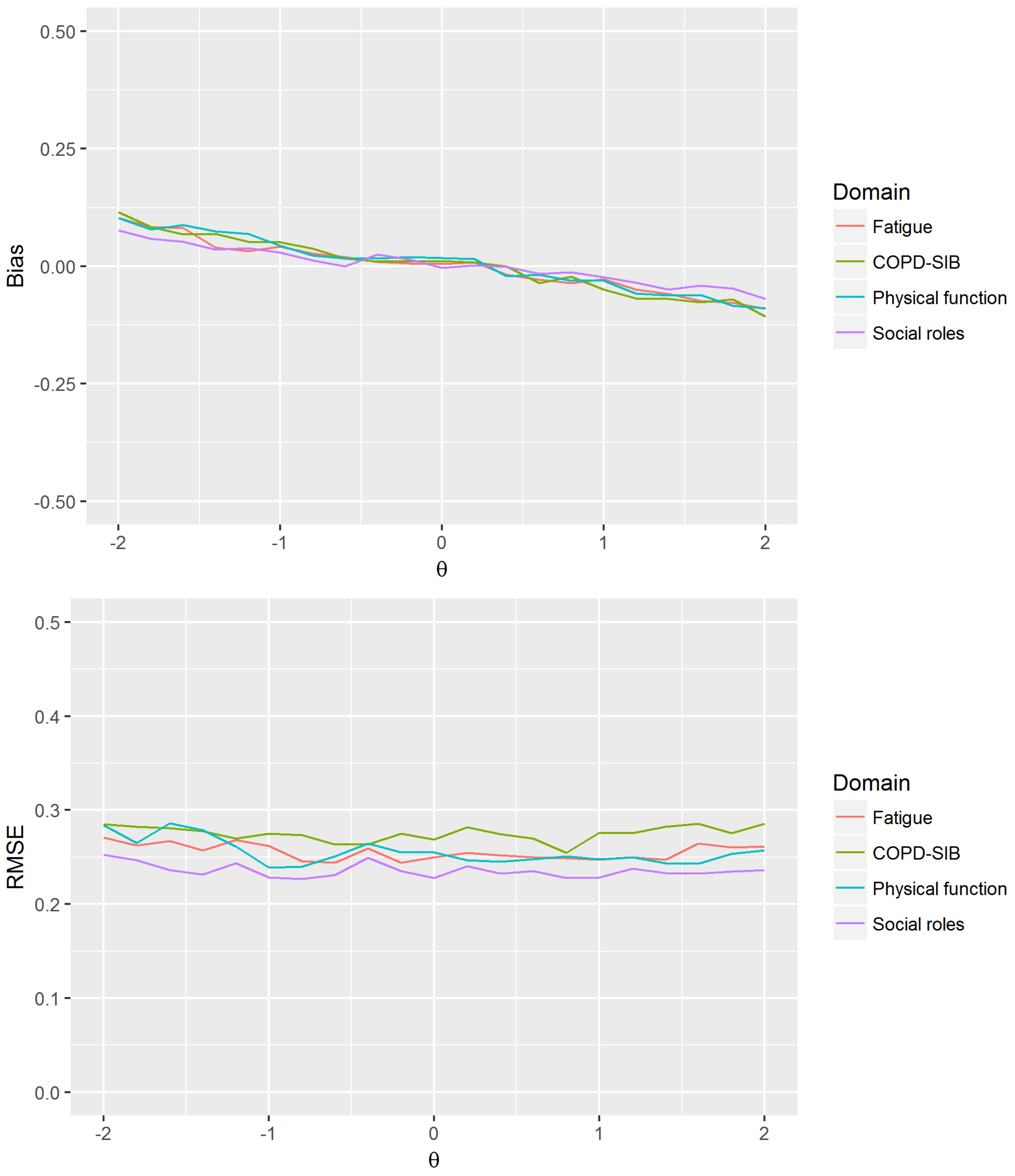

This online supplement accompanies the following paper: *Item usage in a multidimensional computerized adaptive test measuring health-related quality of life* written by Muirne C. S. Paap, Karel Kroeze, Caroline B. Terwee, Job van der Palen, and Bernard P. Veldkamp.

Contact details: Muirne Paap, m.c.s.paap@cemo.uio.no
