# Supplementary material for: Item usage in a multidimensional computerized adaptive test (MCAT) measuring health-related quality of life
Source: Qual Life Res. 2017 Jun 23;26(11):2909–18. doi: 10.1007/s11136-017-1624-3 (PMC5655597; doi:10.1007/s11136-017-1624-3)
Supplement: Supplementary file 4 — Supplementary material 4 (PDF 638 kb) [file 11136_2017_1624_MOESM4_ESM.pdf]

Supplement 4 Item usage rates per dimension

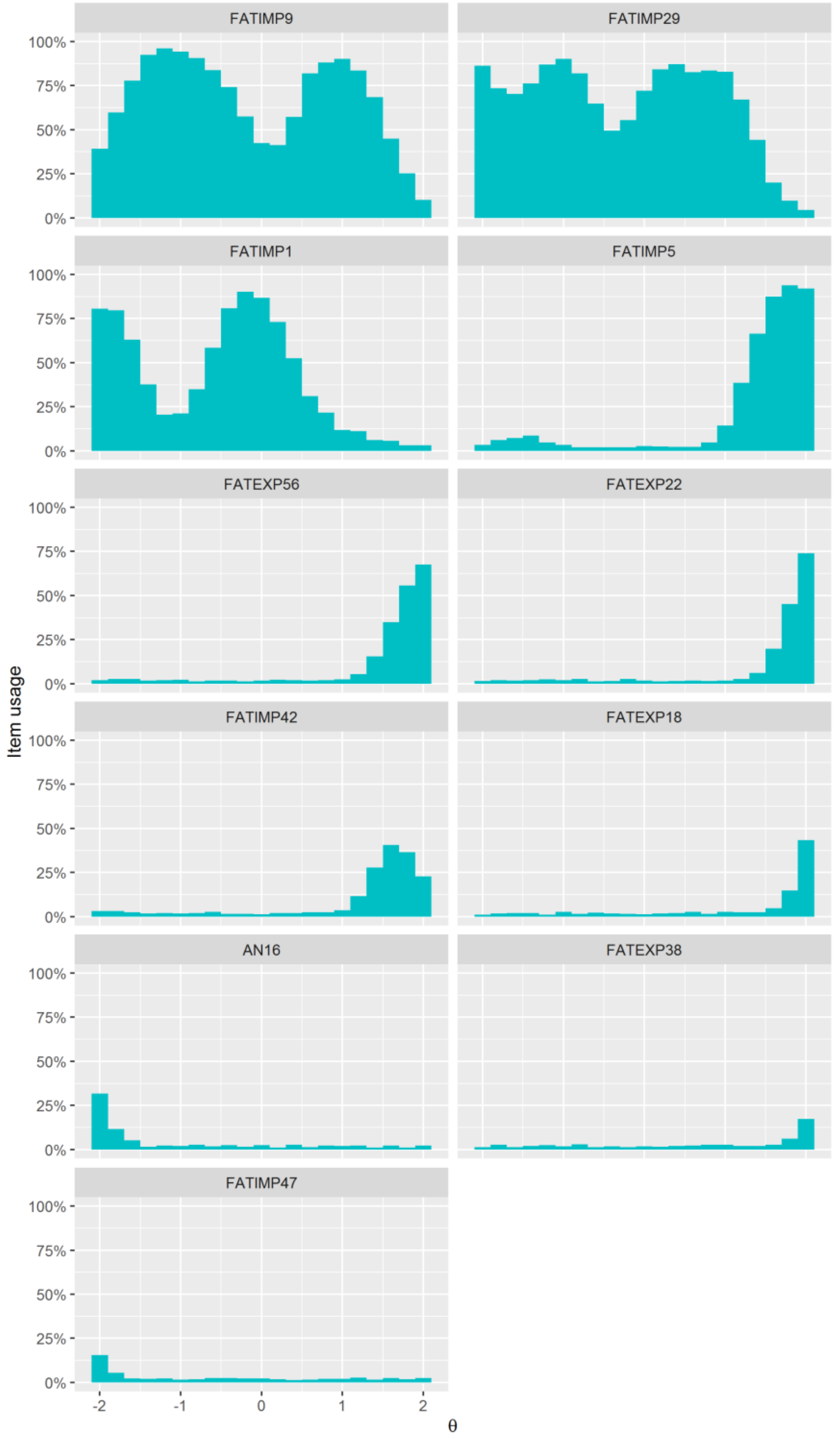

Fig. 1 Item usage rates for *fatigue* items for θ-values between -2 and +2. Only items whose usage rate exceeded the expected usage rate for any θ-value are depicted.

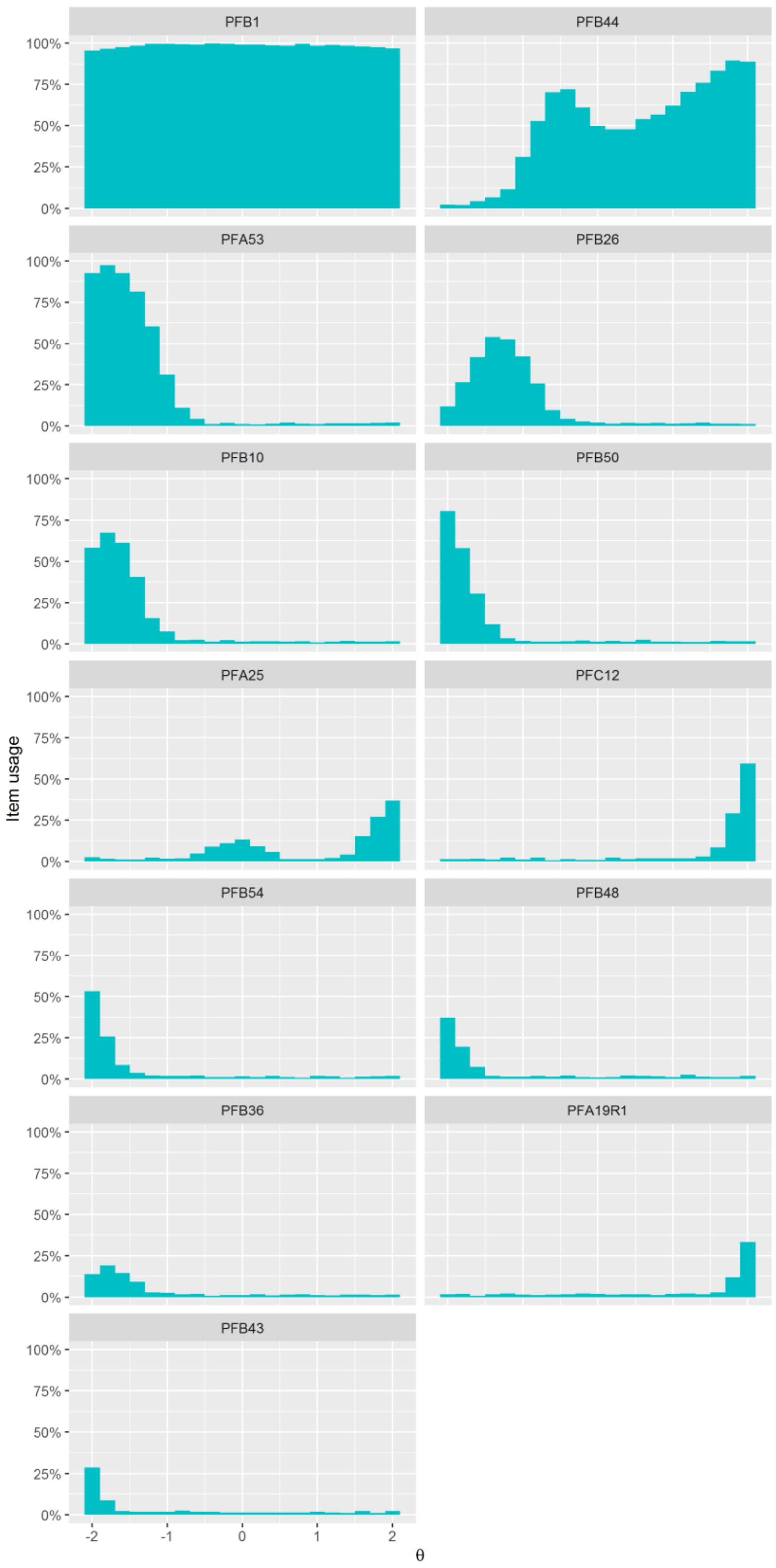

Fig. 2 Item usage rates for *physical function* items for θ-values between -2 and +2. Only items whose usage rate exceeded the expected usage rate for any θ-value are depicted.

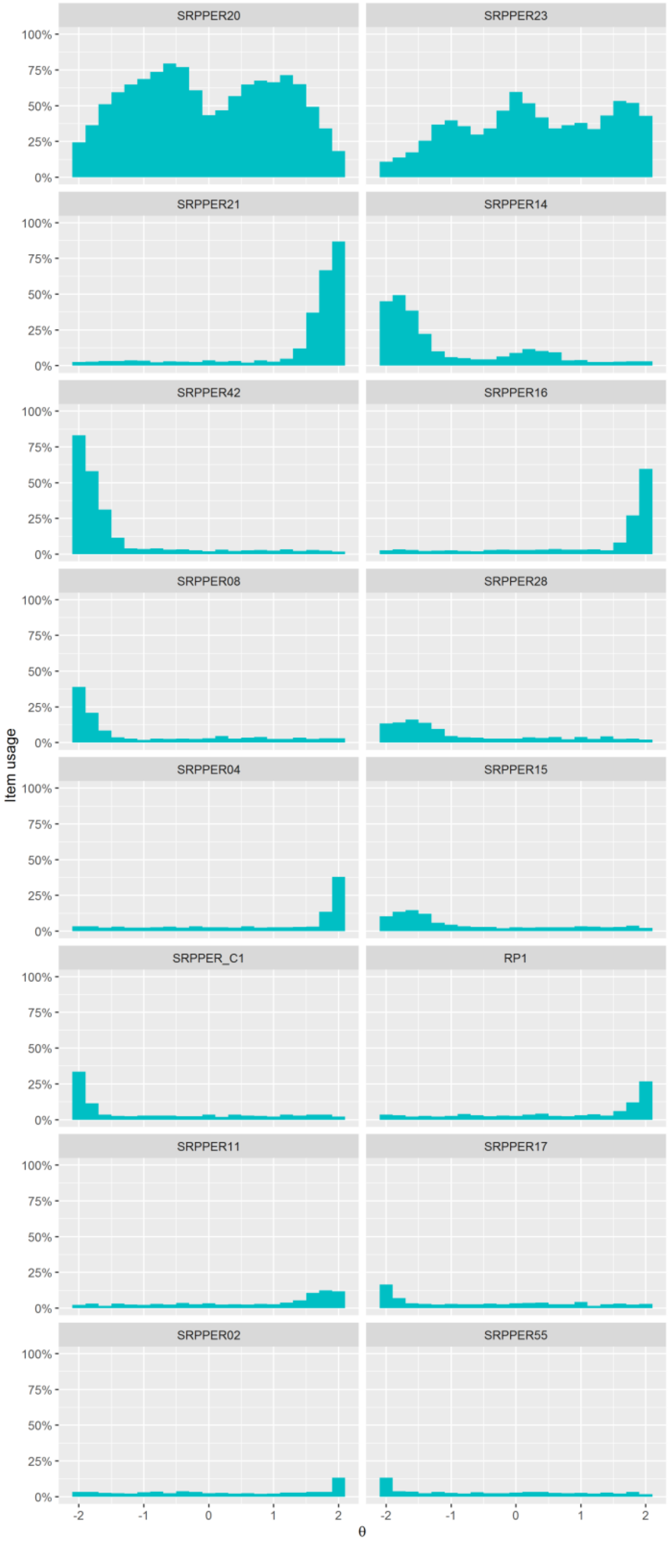

Fig. 3 Item usage rates for *ability to participate in social roles and activities* items for θ-values between -2 and +2. Only items whose usage rate exceeded the expected usage rate for any θ-value are depicted.

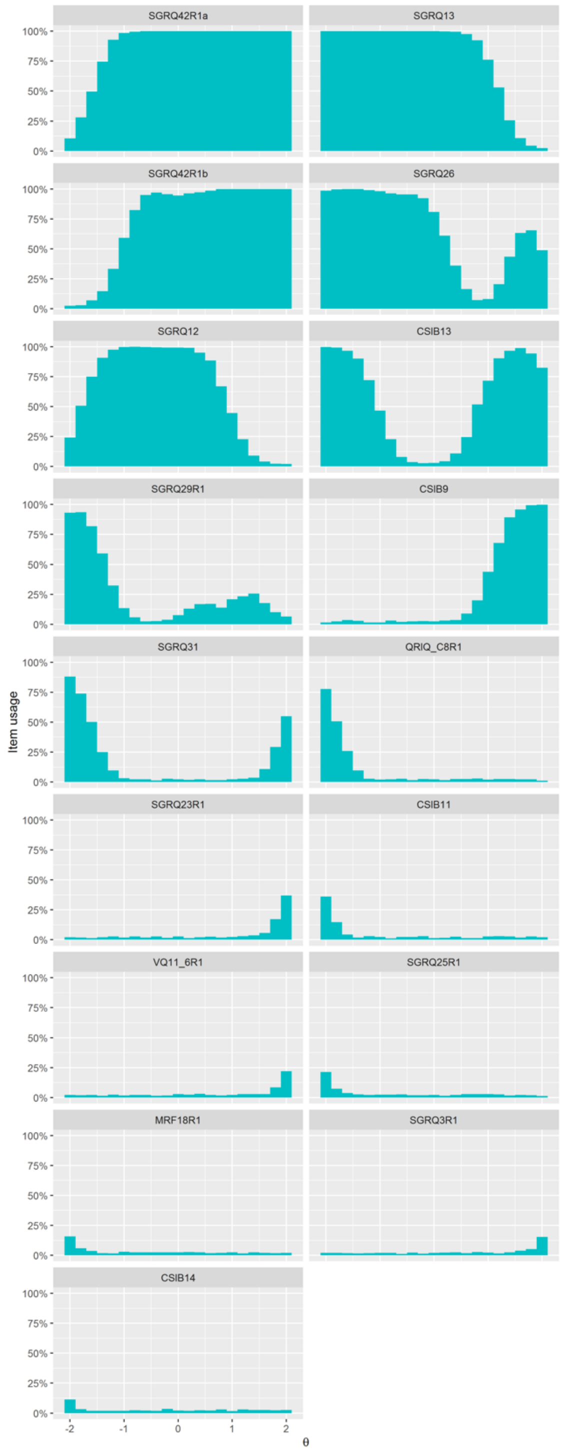

Fig. 4 Item usage rates for *COPD-SIB* items for θ-values between -2 and +2. Only items whose usage rate exceeded the expected usage rate for any θ-value are depicted.

This online supplement accompanies the following paper: *Item usage in a multidimensional computerized adaptive test measuring health-related quality of life* written by Muirne C. S. Paap, Karel Kroeze, Caroline B. Terwee, Job van der Palen, and Bernard P. Veldkamp.

Contact details: Muirne Paap, m.c.s.paap@cemo.uio.no
